# Analyzing temporal dynamics of cell deformation and intracellular movement with video feature aggregation

**DOI:** 10.1186/s12938-019-0638-1

**Published:** 2019-03-01

**Authors:** Fengqian Pang, Zhiwen Liu

**Affiliations:** Department of Information and Electronics, Beijing Institute of Technology, Beijing, 100081 China

**Keywords:** Cell deformation, Intracellular movement, Video feature aggregation, Shape context, SIFT flow

## Abstract

**Background:**

The research and analysis of cellular physiological properties has been an essential approach to studying some biological and biomedical problems. Temporal dynamics of cells therein are used as a quantifiable indicator of cellular response to extracellular cues and physiological stimuli.

**Methods:**

This work presents a novel image-based framework to profile and model the cell dynamics in live-cell videos. In the framework, the cell dynamics between frames are represented as frame-level features from cell deformation and intracellular movement. On the one hand, shape context is introduced to enhance the robustness of measuring the deformation of cellular contours. On the other hand, we employ Scale-Invariant Feature Transform (SIFT) flow to simultaneously construct the complementary movement field and appearance change field for the cytoplasmic streaming. Then, time series modeling is performed on these frame-level features. Specifically, temporal feature aggregation is applied to capture the video-wide temporal evolution of cell dynamics.

**Results:**

Our results demonstrate that the proposed cell dynamic features can effectively capture the cell dynamics in videos. They also prove that the Movement Field and Appearance Change Field Feature (MFAFF) can more precisely model the cytoplasmic streaming. Besides, temporal aggregation of cell dynamic features brings a substantial absolute increase of classification performance.

**Conclusion:**

Experimental results demonstrate that the proposed framework outperforms competing mainstreaming approaches on the aforementioned datasets. Thus, our method has potential for cell dynamics analysis in videos.
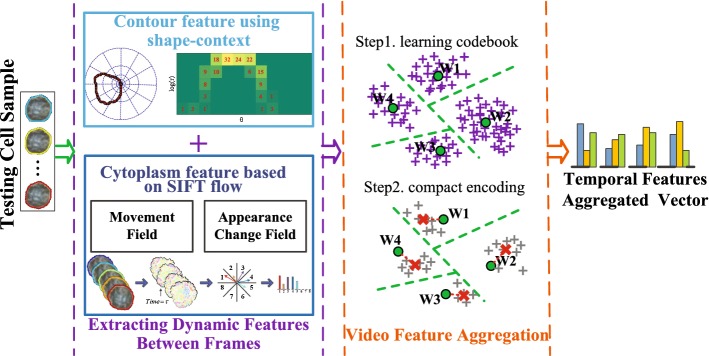

**Electronic supplementary material:**

The online version of this article (10.1186/s12938-019-0638-1) contains supplementary material, which is available to authorized users.

## Background

Image-based cell profiling provides quantitative information about cell state and paves the way to studying biological and biomedical problems [1–4]. As one of most significant aspects therein, characterizing temporal dynamics of cells is used to model cell cycle, analyze migratory phenotypes, and unravel cellular response to physiological stimuli [5–9]. Because of the ability to capture spatio-temporal data, live-cell imaging technology facilitates the analysis of cell dynamics based on image processing and machine learning [10, 11].

To obtain the features for temporal dynamics of cells, cell profiling methods need to precisely characterize the visual appearance of cells and its change on consecutive frames. These methods are divided into two categories according to the cell dynamics they adopted (the deformation of cell contour and the active or directed intracellular movement). Some shape parameters, such as the area or volume, centroid, and circularity, are computed as the global features of cell contour, and the variance of these features is regarded as the index of cell dynamics [5, 12]. However, shape parameters cannot precisely characterize cell morphologies and cell morphology dynamics. The radial distance of cellular contours is employed to preserve more subtle structures of cellular morphology. Similarly, tree graph (TG), a variant of radial distance, is designed for arbitrary cell contours, especially those with cell protrusions [13]. Then the length variation of protrusions (or the number variation of protrusions^1^) between frames is calculated as the feature of cell contour dynamics.

These methods based on the cell contour dynamics make use of some straightforward shape matching strategies. As the accurate correspondence between two cell contours benefits the subsequent deformation measurement, the learning-based shape matching strategy might be a better choices. Shape context measures the shape deformation by optimizing a shape matching problem and is applied to assessing the deformation of anatomical tissue and falling human silhouettes [14–16]. Thus it is suitable to be introduced into our framework to quantize the deformation of cellular contours.

Another category of cell dynamics, or alternatively, the intracellular movement is also relevant to the cell dynamics. Image cross-correlation is employed to obtain time-dependent speckle pattern derived from optical coherence microscopy images as the representation of the cell dynamics [17]. Furthermore, cytoplasmic streaming is modeled to construct the movement field (or displacement field) between a pair of frames based on optical flow, and then the average horizontal velocity and vertical velocity are concatenated into the feature vector according to temporal order [18]. In fact, besides intracellular movement, there is the phenomenon of splitting, merging and disappearing of them during cytoplasmic streaming [19]. And this phenomenon corresponds to the change of intensity or texture around the moving particles, i.e., the changes of image local properties. Hence, it is reasonable to construct an additional appearance change field for cytoplasmic streaming as a complement to the original movement field.

Nevertheless, optical flow is based on the brightness constancy assumption, which cannot construct a meaningful appearance change field and is sensitive to the variance of light, perspective, and noise. Scale-Invariant Feature Transform (SIFT) flow can obtain the robust semantic-level correspondence between two images. In this paper, we employ SIFT flow to establish the movement field and appearance change field for cytoplasmic streaming. Herein the appearance change field is constructed by computing the discrepancy of the corresponding SIFT descriptors.

Although the aforementioned methods successfully capture the cell dynamics from short-term video segments, the subsequent video-range aggregation of these features is not considered in-depth. They only adopt the concatenation or accumulation strategy for the features along temporal dimension [18, 20–22]. To preserve more temporal structures, hidden Markov models (HMM) are introduced to represent the cell shape dynamics in time series as predefined morphological states. This process condenses the temporal dynamics into a simpler representation, which enhances discriminative power for profiling temporal dynamics [23]. Therefore, HMM is applied to recognize the cellular phases during mitosis and cellular-response-based drug classification [6, 24]. Similarly, in the previous work of this study, a temporal bag of words (TBoW) model is utilized to fuse cell dynamic features between frames [25]. The TBoW model learns a codebook containing the typical modes of cell short-term dynamics and encodes these short-term dynamics as visual words in a codebook. Finally, the word frequency of the codebook is defined as the video-range cell dynamics.

HMM and TBoW only transform the primary features into predefined states (or visual words) or sample statistics, i.e., the number of states. Compact encoding, by contrast, exploits more statistics, such as mean, variance, as well as even skewness and kurtosis, which leads to its great advantage over HMM and TBoW. In this paper, we introduce the compact encoding for the sake of modeling the temporal dynamics in live-cell videos. We further compare Fisher vector (FV) [26, 27], vector of locally aggregated descriptors (VLAD) [28, 29] and higher-order VLAD (H-VLAD) [30] to find out the best one for our application.

**Fig. 1:**
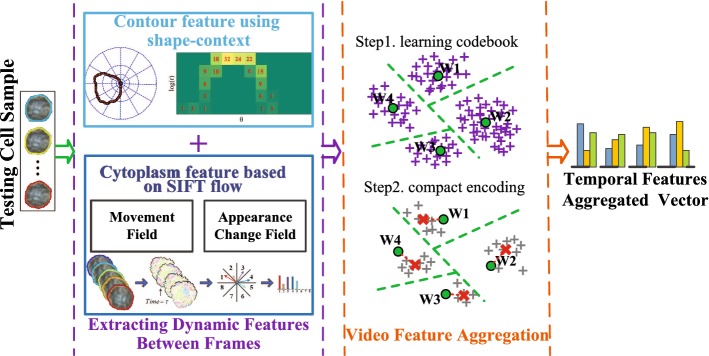
The pipeline of the proposed video feature aggregation (VFA) to analyze temporal dynamics of cells. Cell dynamic features are extracted between frames, which contains contour feature and cytoplasm feature. Then, a VFA is applied based on these cell dynamic features

This paper mainly proposes a novel framework to evaluate temporal dynamics of cells as shown in Fig. 1, and its contributions are threefold. First, shape context is introduced to measure the deformation of cellular contours. Second, SIFT flow is utilized to model the complementary movement field and appearance change field for the cytoplasmic streaming simultaneously. Finally, we introduce and compare three mainstreaming compact encoding approaches to temporal aggregation of dynamic features, and discover the most suitable encoding strategy for the whole framework.

## Materials and methods

In this section, we first describe two live-cell video datasets for evaluating the utility of the proposed framework. These two datasets individually contain 80 video clips in two classes and 120 video clips in four classes. Then the cell dynamic features between frames are presented, which include the contour feature using shape-context and the cytoplasm feature based on SIFT flow. Finally, we present the temporal aggregation strategy for these dynamic features to generate the video-wide representation.

### Data

To validate the proposed approach, there are two datasets of video clips about lymphocytes established by the collaboration hospital, Beijing You’an Hospital. The lymphocytes were from the blood samples that were collected from the tails of the mice (6–8 weeks, 20–22 g) after the skin transplantation, and the video clips (20–30 s, 25 frames per second, 288 × 352 video resolution, AVI format) were recorded with the phase contrast microscopy (Olympus BX51) at a magnification of 1000. After the video clips were obtained, they are further enlarged 16 times by up-sampling. Each time only one target lymphocyte was observed and manually positioned in the center of the field. Then a quality control step was conducted beforehand to filter out the video clips containing only one lymphocyte. And it also guarantees that there is no overlap and trajectory cross between the lymphocyte and red blood cells. There are two types of skin transplantation, i.e., the self-skin transplantation group (SST group) and the allergenic-skin transplantation group (AST group). In the SST group, a healthy Balb/C male mouse was used as both the host and the donor, while in the AST group a pair of healthy Balb/C male mouse and healthy C57BL/6 male mouse were used as the host and the donor, respectively. Several video clips of the datasets are available at http://isip.bit.edu.cn/kyxz/xzlw/77051.htm.

For Dataset I, there are 80 video clips in total (40 video clips for each class) obtained from a contrast experiment, in which both the SST group and AST group have 20 hosts and 20 donors. On the fourteenth day after the surgery, the lymphocytes in Dataset I were obtained from the blood samples collected from the tail vein. The lymphocytes in the second group showed irregular dynamic behavior, such as the cell elongation from different angles and the obvious movement of intracellular cargoes compared with the first group. Consequently, the videos from the first group and the second group were categorized as normal and abnormal, respectively.

Dataset II is composed of 120 video clips equally divided into four classes, which is derived from an AST group experiment with 25 pairs of hosts and donors. On the seventh day after the skin transplantation, the lymphocytes of Dataset II were obtained from the blood samples collected from the tail vein. The videos were divided into four classes (normal, slight activation, moderate activation, and drastic activation) according to the cellular deformation by three experts with a voting protocol. For these two datasets, we use 30 random splits of the data, while considering 20 random video clips per class for training and the rest for testing.

**Fig. 2:**
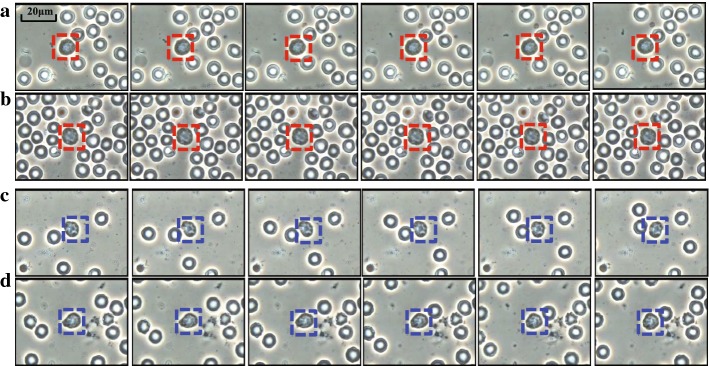
Samples from live-cell video datasets arranged in rows. The frames in different columns are extracted from certain videos at the fixed time interval of 3 s. Only one target lymphocyte (in the red/blue dashed box) is observed once, and there is no overlap and trajectory cross between the lymphocyte and red blood cells. The frames of the samples in **a** and **b** are both from the SST group in Dataset I, while those in **c** and **d** from the AST group in Dataset I

### Preprocessing

For the effectiveness of feature extraction, some pre-processing procedures need to be adopted, containing cell segmentation and cell tracking in each frame, as well as cell alignment among the sequence of frames. In Fig. 2, each row corresponds to a video clip in the datasets in “Data” section, and the target cells are lymphocytes in the red/blue dashed box. We employ an active contour model designed for live cells in phase-contrast images to automatically segment and track cells [31]. To further eliminate the impact of compulsory movement, video stabilization algorithm is introduced to perform a non-rigid alignment for the cell sequences in frames [32]. Besides, manual validation is exploited to eliminate the ambiguity of cell segmentation and tracking by human eye if necessary. In detail, we can specified the initial contours for the lymphocytes to make sure the accuracy of cell segmentation.

### Dynamic features between frames

This subsection mainly describes the features of cell dynamics between frames in the image sequence. Specifically, the dynamic features can be extracted from the deformation of cellular contours and intracellular movements. The former is captured by the shape context while the latter is modeled with SIFT flow. Then the corresponding contour feature and cytoplasm feature are combined to form a robust feature vector of cell dynamics.

#### Contour feature using shape-context

In the field of object recognition and shape matching, shape context was first proposed by [14], and then has been widely used in digit recognition, trademark search, and image registration. Shape context is introduced into the framework of deformation assessment for anatomical tissue to preserve and discriminate tiny deformation [15]. While shape context also has the potential to match the silhouettes of the falling human body and take the mean matching cost as a crucial index to quantify the deformation [16]. Therefore, this paper adopts shape context for the sake of generating the cellular contour deformation feature.^2^

#### Shape context

**Fig. 3:**
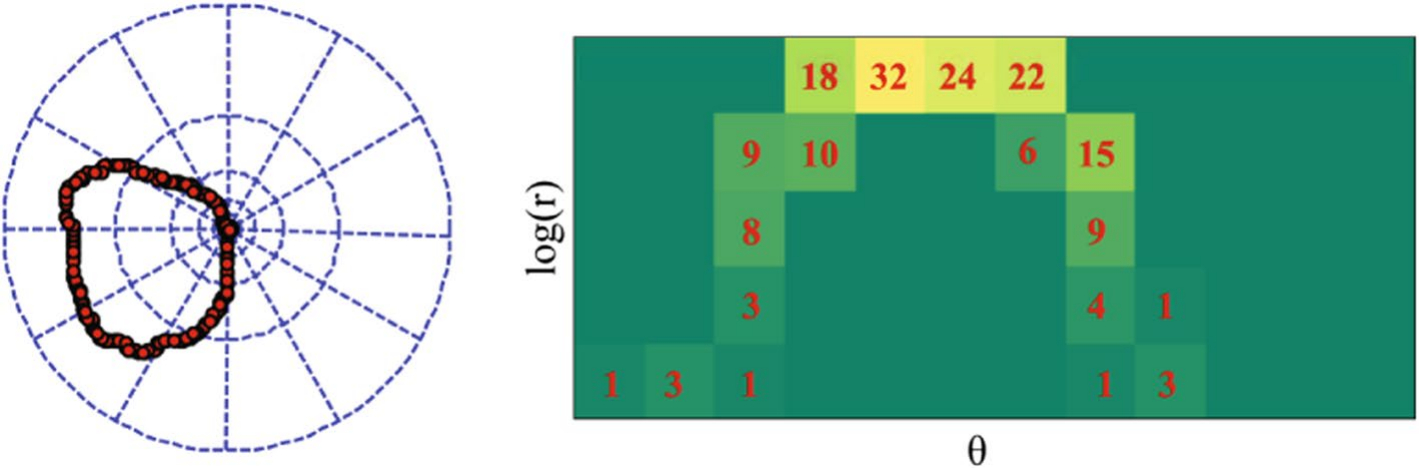
Log-polar histogram computation for a point $p_{i}$. The log-polar histogram has 5 bins for $log\, r$ and 12 bins for $\theta$ as proposed by [14]

In shape context, a shape is sampled into a discrete set of points from its contour, which will finally accumulate a log-polar histogram $h_{i}$:

1 $$\begin{aligned} h_{i}(k) = \#\{q\ne p_{i}:\,(q-p_{i})\in bin(k)\}, \end{aligned}$$

where $p_{i}$ is a point in the given *n*-points shape, and its shape context $h_{i}$ records the relative coordinates of the remaining $n-1$ points as shown in Fig. 3. *bin*(*k*) stands for the *k*-th bin in histogram $h_{i}$. Suppose $p_{i}$ and $q_{j}$ are from two shapes *P* and *Q*, respectively, therefore the matching cost $C_{ij}$ for each pair of points $(p_{i},\, q_{j})$ is computed with the $\chi ^{2}$ statistic:

2 $$\begin{aligned} C_{ij} = \frac{1}{2}\sum _{k=1}^{K}\frac{\left[ h_{i}(k)-h_{j}(k)\right] ^{2}}{h_{i}(k)+h_{j}(k)}, \end{aligned}$$

where $h_{i}(k)$ and $h_{j}(k)$ denote the *K*-bin histograms for $p_{i}$ and $q_{j}$, separately.

#### Contour feature based on shape distance

Hungarian algorithm [33] can find the best matching by minimizing the total cost $H(\pi )=\sum C(p_{i},\, q_{\pi (i)})$ given a permutation $\pi (i)$. With the permutation, a series of transformations $T=\left\{ T_{k}\right\} _{k=1\ldots u}$ for each point can be computed using the thin plate spline model (TPS). Then several iterations of shape context matching and TPS re-estimation are implemented, and the shape context distances $D_{sc}^{1}, \ldots , D_{sc}^{L}$ in *L* iterations are concatenated as the feature vector of cellular contour deformation $F_{DCS}=\{D_{sc}^{1}, \ldots ,\, D_{sc}^{l}, \ldots , D_{sc}^{L}\}$.

3 $$\begin{aligned} D_{SC}^{l}=\frac{1}{n}\sum _{p\in P}arg\underset{{\scriptstyle q\in Q}}{min}C(p,T(q))+\frac{1}{m}\sum _{q\in Q}arg\underset{{\scriptstyle p\in P}}{min}C(p,T(q)), \end{aligned}$$

where $T(\cdot )$ denotes the TPS shape transformation.

#### Cytoplasm feature based on SIFT flow

In SIFT flow, the SIFT descriptor, as a type of middle-level representation, is incorporated into the computational framework of optical flow. It establishes a robust semantic-level correspondence through matching these image structure [34].^3^ Based on the semantic-level correspondence, the movement field and the appearance change field are constructed by computing the displacement of the corresponding points and the discrepancy of the corresponding SIFT descriptors, respectively. Then histograms of oriented SIFT flow is employed to characterize multi-oriented dynamic information from both the movement field and the appearance change field.

#### SIFT flow

Instead of matching raw pixels in optical flow, SIFT flow searches for the correspondences of SIFT descriptors on the grid coordinate $p=(x,\, y)$ of images. The dense correspondence map, or the movement field, can be obtained by minimizing an objective function *E*(*w*):

4 $$\begin{aligned} E(w)&=\sum _{p}min\left( \left\| s_{p}^{1}-s_{p+w_{p}}^{2}\right\| _{1},\, t\right) +\sum \eta \left( \left| u_{p}\right| +\left| v_{p}\right| \right) \nonumber \\&\quad +\sum _{(p,q)\in \varepsilon }min\left( \alpha \left| w_{p}-w_{q}\right| ,\, d\right) , \end{aligned}$$

where $s_{p}^{1}$ and $s_{p}^{2}$ individually denote the SIFT descriptor at position *p* in two SIFT images, and $w_{p}=(u_{p},\, v_{p})$ presents the flow vector at *p*. The parameters *t* and *d* are the thresholds of the data term and the smoothness term, respectively. The set $\varepsilon$ contains all spatial four-neighborhoods.

After obtaining the correspondence map upon the sequential SIFT images, the appearance change field can be implemented by computing the difference of SIFT features between the corresponding points.

**Fig. 4:**
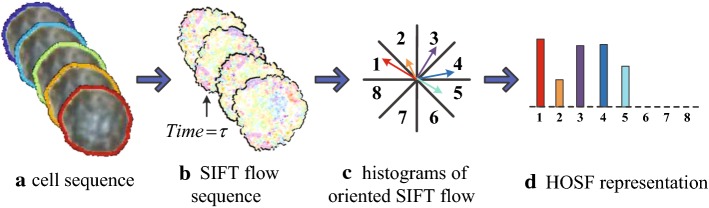
The illustration for histograms of oriented SIFT flow (HOSF). After constructing the movement field (or the appearance change field), the constructed field is binned in terms of their primary angle from the horizontal axis and weighted according to its magnitude (or appearance difference)

#### Histograms of oriented SIFT flow

Due to the susceptibility to scale changes and directionality of movement, the raw SIFT flow cannot obtain a good performance if applied as features directly. Inspired by the histograms of oriented optical flow, SIFT flow is binned according to its primary angle from the horizontal axis and weighted according to its magnitude or appearance difference, as shown in Fig. 4. $F_{MDF}=\left\{ f_{MDF}^{1},\,\ldots ,\, f_{MDF}^{R}\right\}$ and $F_{ACF}=\left\{ f_{ACF}^{1},\,\ldots ,\, f_{ACF}^{R}\right\}$ are obtained to characterize the movement and appearance variation of the cytoplasm, respectively.

5 $$\begin{aligned} f_{MDF}^{r}= & {} \sum _{u,v\in bin(r)}\sqrt{u_{p}^{2}+v_{q}^{2}}, \end{aligned}$$

6 $$\begin{aligned} f_{ACF}^{r}= & {} \sum _{u,v\in bin(r)}\left\| s_{p}^{1}-s_{p}^{2}\right\| , \end{aligned}$$

where $f_{MDF}^{r}$ denotes the accumulation of displacement magnitude belonging to the *r*-th ($1\le r\le R$) bin in the movement field, and $f_{ACF}^{r}$ means the sum of the appearance difference in the *r*-th ($1\le r\le R$) bin.

#### Combination of features

The robustness of feature representation can be enhanced by combining the complementary features. To sum up, the aforementioned $F_{DCS}$, $F_{MDF}$ and $F_{ACF}$ between frames are concatenated to form a feature vector:

7 $$\begin{aligned} F_{i}=\left\{ F_{DCF},\, F_{MDF},\, F_{ACF}\right\} . \end{aligned}$$

The computing of $F_{i}$ is the key step to extracting the features of cell dynamics in the whole framework. Then “Temporal aggregation of dynamic features” section is mainly about encoding the chronological structure of the cell dynamic features in a particular video.

**Fig. 5:**
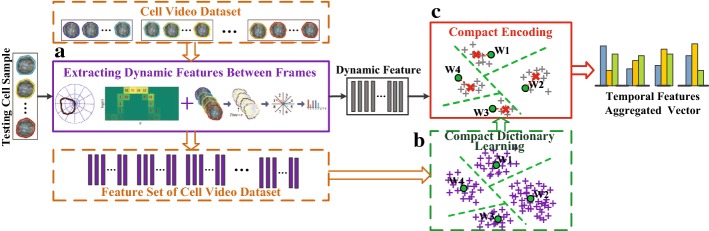
The illustration of temporal aggregation for dynamic features. Module A is responsible for extracting the frame-level dynamic feature for the training dataset or testing sample. And Module B plays a part in the training phase to generate a compact dictionary. Based on the compact dictionary, Module C encodes the dynamic features from the module A into the final video level representation

### Temporal aggregation of dynamic features

For the video-wide cell dynamics, it is essential to aggregate a series of frame-level dynamic features along temporal extent in a rational way. That is to say, it needs to consider how dynamic features evolve over time in a video. In this section, we present three compact encoding methods, including FV, VLAD, and H-VLAD, to capture the temporal information of cell sequences. The pipeline for the temporal aggregation strategy in this paper is depicted in Fig. 5. It can be summarized as the following two phases: (1) In the training phase, the samples in the cell video dataset are transformed into the dynamic features by the aid of algorithms in “Dynamic features between frames” section. Then the compact dictionary with *K* visual words is learned based on these features by means of K-Means or Gaussian mixture model (GMM). (2) In the testing phase, the features of cell dynamics are obtained similarly and assigned to the *K* visual words. Then, the residuals between the visual words and the dynamic features belonging to them are encoded into the temporal-feature-aggregated vector.

#### Fisher vector encoding

In FV encoding [26, 27], a GMM with *K* components can be learned from the training dynamic features between frames, and denoted as $\varTheta =\left\{ (\varvec{\mu }_{k},\varvec{\sigma }_{k},\pi _{k}),\, k=1,2,\ldots ,K\right\}$, where $\varvec{\mu }_{k}$, $\varvec{\sigma }_{k}$, $\pi _{k}$ are the mean vector, variance matrix (assumed diagonal) and mixture weight of the *k*-th component, respectively. Given ${\varvec{X}}=({\varvec{x}}_{1},{\varvec{x}}_{2}\ldots \,,{\varvec{x}}_{N})$ of dynamic features extracted from a testing cell image sequence, we have mean and covariance deviation vectors for the *k*-th component as:

8 $$\begin{aligned} {\varvec{u}}_{k}&=\frac{1}{N\sqrt{\pi _{k}}}\sum _{i=1}^{N}q_{ki}\left( \frac{{\varvec{x}}_{i}-\varvec{\mu }_{k}}{\varvec{\sigma }_{k}}\right) ,\nonumber \\ {\varvec{v}}_{k}&=\frac{1}{N\sqrt{2\pi _{k}}}\sum _{i=1}^{N}q_{ki}\left[ \left( \frac{{\varvec{x}}_{i}-\varvec{\mu }_{k}}{\varvec{\sigma }_{k}}\right) ^{2}-1\right] , \end{aligned}$$

where $q_{ik}$ is the soft assignment of feature ${\varvec{x}}_{i}$ to the *k*-th Gaussian component. By concatenation of ${\varvec{u}}_{k}$ and ${\varvec{v}}_{k}$ of all the *K* components, FV for the testing sample is formed with size $2D^{'}K$, where $D^{'}$ is the dimension of the dynamic feature after principal component analysis (PCA) pre-processing [27]. Power normalization using signed square root (SSR) with $z=sign(z)\sqrt{\left| z\right| }$ and $\ell _{2}$ normalization are then applied to the FVs [26, 27].

#### VLAD encoding

As a non-probabilistic version of FV encoding, VLAD encoding [28, 29] simply utilizes K-means instead of GMM to generate *K* coarse centers $\left\{ {\varvec{c}}_{1},{\varvec{c}}_{2},\ldots \,,{\varvec{c}}_{K}\right\}$. Then we can obtain the difference vector ${\varvec{u}}_{k}$ with respective to the *k*-th center ${\varvec{c}}_{k}$ for the testing dynamic feature set by:

9 $$\begin{aligned} {\varvec{u}}_{k}=\sum _{i:NN({\varvec{x}}_{i})={\varvec{c}}_{k}}\left( {\varvec{x}}_{i}-{\varvec{c}}_{k}\right) , \end{aligned}$$

where $NN({\varvec{x}}_{i})$ indicates ${\varvec{x}}_{i}$’s nearest neighbors among *K* coarse centers.

The VLAD encoding vector concatenates ${\varvec{u}}_{k}$ over all the *K* centers with size $D^{'}K$, and the post-processing employs the power and $\ell _{2}$ normalization. Besides, the intra-normalization [35] is also applied to add normalization on each ${\varvec{u}}_{k}$. The proposed framework prefer to VLAD-*k* (*k* = 5), a variant of VLAD, which extends the nearest neighbor with the *k*-nearest neighbors, because of its good performance in contrast to the original VLAD [36].

#### High-order VLAD encoding

In order to keep both high performance and high extraction speed, the H-VLAD [30] augments the original VLAD with high-order statistics, e.g., diagonal covariance and skewness. The *K* clusters are first learned by K-means, regarded as the visual words $\left\{ {\varvec{w}}_{1},{\varvec{w}}_{1},\ldots \,,{\varvec{w}}_{K}\right\}$, and the corresponding first-order, second-order, and third-order statistics are denoted as $\left\{ \varvec{\mu }_{1},\varvec{\mu }_{2},\ldots \,,\varvec{\mu }_{K}\right\}$, $\left\{ \varvec{\sigma }_{1},\varvec{\sigma }_{2},\ldots \,,\varvec{\sigma }_{K}\right\}$ and $\left\{ \varvec{\gamma }_{1},\varvec{\gamma }_{2},\ldots \,,\varvec{\gamma }_{K}\right\}$, respectively. The technical details of H-VLAD can be summarized as:

10 $$\begin{aligned} {\varvec{u}}_{k}&=N_{k}\left( \frac{1}{N_{k}}\sum _{i=1}^{N_{k}}{\varvec{x}}_{i}-\varvec{\mu }_{k}\right) =N_{k}({\varvec{m}}_{k}-\varvec{\mu }_{k}),\nonumber \\ {\varvec{v}}_{k}&=\frac{1}{N_{k}}\sum _{i=1}^{N_{k}}\left( {\varvec{x}}_{i}-{\varvec{m}}_{k}\right) ^{2}-\varvec{\sigma }_{k}^{2},\nonumber \\ {\varvec{s}}_{k}&=\frac{\frac{1}{N_{k}}\sum _{i=1}^{N_{k}}\left( {\varvec{x}}_{i}-{\varvec{m}}_{k}\right) ^{3}}{\left( \frac{1}{N_{k}}\sum _{i=1}^{N_{k}}\left( {\varvec{x}}_{i}-{\varvec{m}}_{k}\right) ^{2}\right) ^{\frac{3}{2}}}-\varvec{\gamma }_{k}, \end{aligned}$$

where ${\varvec{X}}_{k}=\left\{ {\varvec{x}}_{1},{\varvec{x}}_{2},\ldots \,,{\varvec{x}}_{N_{k}}\right\}$ is the testing dynamic features belonging to the *k*-th visual word ${\varvec{w}}_{k}$, and ${\varvec{m}}_{k}$ stands for the mean of these dynamic features. Therefore, ${\varvec{u}}_{k}$, ${\varvec{v}}_{k}$ and ${\varvec{s}}_{k}$ are the residual vectors of the first-order, second-order and third-order, respectively. Similar to the original VLAD, the final representation of H-VLAD is concatenated as $\left\{ {\varvec{u}}_{1},{\varvec{v}}_{1},{\varvec{s}}_{1},{\varvec{u}}_{2},{\varvec{v}}_{2},{\varvec{s}}_{2}\ldots \,,{\varvec{u}}_{K},{\varvec{v}}_{K},{\varvec{s}}_{K}\right\}$, and the post-processing operation also adopts the power-, $\ell _{2}$- and intra-normalization [35].

#### Analysis of temporal feature aggregation methods

**Fig. 6:**
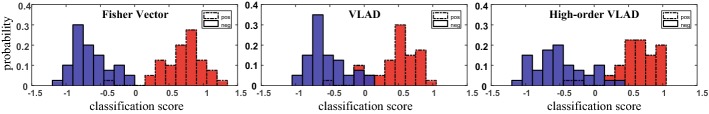
Histogram distribution of classification scores from positive exemplars (blue and plain) and negative exemplars (red and dashed). The purple area stands for the overlap of positive histogram and negative histogram. Among these three encoding methods, FV encoding with the smallest overlap implies it has the best discrimination

Given the above three compact encoding approaches to temporal feature aggregation, we need to find out which one is the most appropriate for our application. For this purpose, we conduct an experiment on Dataset I (for details see “Data” section ) to analyze the discrimination of these encoding strategies: FV, VLAD, and H-VLAD. Specifically, we calculate the histogram distribution of classification scores from positive exemplars and negative exemplars, respectively. The positive and negative exemplars individually correspond to 20 training samples from SST group and the AST group. Note that the classifier is the linear SVM (the parameters are the same as “Experimental setup” section ), the dictionary size is 64 for FV, VLAD, and H-VLAD, and the encoding vector is not followed by the temporal pyramid pooling (TPP). From Fig. 6, we can find that VLAD encoding and H-VLAD encoding have the similar discrimination while the FV encoding has better performance. It shows that the FV encoding is most suitable for the temporal aggregation of cell dynamic features.

#### Temporal pyramid pooling

To preserve much more temporal discrimination, we add the TPP, regarded as a one-dimensional version of spatial pyramid pooling [37]. For a particular video, we suppose that its dynamic features between frames is denoted as ${\varvec{Z}}$ and the temporal aggregation operation is defined as ${\varPhi }(\cdot )$. TPP is to organize the dynamic features ${\varvec{Z}}$ into three level of subsets: $Z_{1}^{1}$, $Z_{2}^{1}$, $Z_{2}^{2}$, $Z_{3}^{1}$, $Z_{3}^{2}$ and $Z_{3}^{3}$, which have 1, 2 and 3 average-partitioned subwindows along temporal dimension, respectively. Therefore, the TPP of ${\varvec{Z}}$ can be written as follows:

11 $$\begin{aligned} \varPhi ({\varvec{Z}})=[\varPhi (Z_{1}^{1}),\,\varPhi (Z_{2}^{1}),\,\varPhi (Z_{2}^{2}),\,\varPhi (Z_{3}^{1}),\,\varPhi (Z_{3}^{2}),\,\varPhi (Z_{3}^{3})]. \end{aligned}$$

## Experimental results

In this section, we present a detailed experimental evaluation of our proposed framework based on the cell-video datasets in “Data” section. Several exploration experiments were conducted to determine the crucial parameters of the proposed approach. Moreover, the proposed approach is compared with several existing methods.

### Experimental setup

**Fig. 7:**
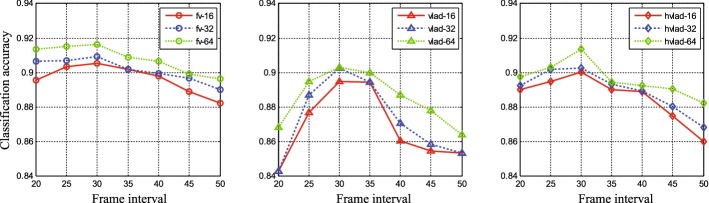
The contrast experiment about the performance with different frame intervals. Three figures from left to right show the classification accuracy of FV, VLAD and H-VLAD on DataSet I respectively. And the green dashed line, blue dashed line and red plain line are corresponding to different vocabulary size (16, 32, 64)

The parameters used in our approach can be divided into three parts: the parameters in feature extraction, feature encoding, and classifier. Firstly, both shape-context for contour deformation and SIFT flow for cytoplasmic streaming adopt the default parameters as reported in the literatures [14, 34]. And the number of bins in histograms of oriented SIFT flow is setting to 36. As the frame interval has direct relationship with cell dynamics, we conduct a contrast experiment about different frame intervals. As shown in Fig. 7, it is able to achieve the best performance with different encoding methods or based on various vocabulary sizes, when the frame interval equals 30 (as default value without assignment) (Additional file 1: Figure S1). Secondly, there is an important parameter, vocabulary size, which is related to not only the encoding discrimination but also the classifier overfitting (the discussion in “Performance evaluation of temporal aggregation” section). Finally, the linear classifier in LibSVM toolkit [38] is adopted. After the parameters are chosen, we retrain the classifier based on the 30 random splits of two Dataset (refer to “Data” section), and the penalty coefficient is determined on the training set using fivefold cross-validation.

### Validation of dynamic features between frames

In this paper, the frame-level dynamic features are extracted in two aspects: contour deformation and cytoplasmic streaming. Based on Dataset II, we first extract various kinds of dynamic features between frames, which contain Zernike moment (ZM) [7], TG [13], radial-distance feature (RDF) [20], shape-context feature (SCF), FV (OFF) [18], SIFT flow feature (SFF), the complementary Movement Field and Appearance change Field Feature (MFAFF), and the combined feature vector (CF) in “Combination of features” section. For a fair comparison, we make the dimension of all the frame-level dynamic features maintain 30 by choosing appropriate parameters for each feature. ZM with 30 orders is captured from the samples. TG and RDF are both sampled into 30 discrete points. TG samples the perimeter of the cell contour according to equal interval strategy, while RDF is based on equal angle-interval sampling principle. The number of iterations *L* for SCF is specified as 30. The dimensions of OFF, SFF and MFAFF are decided by the histogram of oriented optical/SIFT flow, i.e. the number of histogram bins *R*. In detail, the *R* for OFF and SFF is set to 30, while the *R* for MFAFF is chosen as 15. CF is the combination of SCF and MFAFF, thus *L* and *R* are both set to 10. Then for all kinds of dynamic features, a video-range aggregation strategy is implemented by the average pooling, i.e., averaging frame-level features of each video along the temporal dimension. At last, we perform the classification with the aggregated features using SVM.

**Fig. 8:**
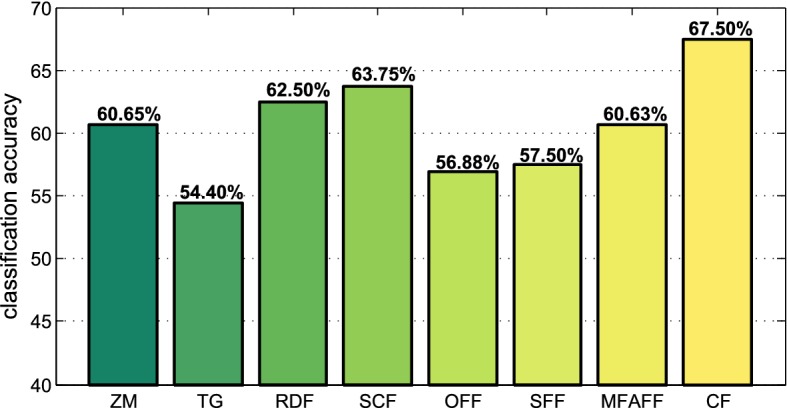
Comparison of cell dynamic features. For contour deformation, SCF is better than ZM, TG and RDF. With respect to the cytoplasmic streaming, SFF also achieves better performance comparing with OFF. At last, the CF reaches the highest classification accuracy

As shown in Fig. 8, SCF achieves a better performance than ZM, TG, and RDF, which proves the effectiveness of our proposed contour feature. TG and RDF both belong to the radial-distance-based feature, but TG obtains 8.1% lower accuracy than RDF. The reason might be that TG is designed for the dynamics of cell protrusions and lymphocytes in our dataset does not have the explicit protrusions. In the aspect of cytoplasm motion features, SFF achieves 0.625% higher accuracy than OFF. Moreover, the features from the MFAFF further improve the performance in contrast to SFF. Comparing the dynamic features in two aspects, we can find out that the contour deformation features play a more dominant role in the process of characterizing cell dynamics. Finally, the CF reaches the highest classification accuracy (67.50%), which illustrates the significance of combining the cellular contour and cytoplasm streaming dynamics.

**Table 1:** Classification result (accuracy in percentage) of FV with different vocabulary sizes

	Normal	Slight activation	Moderate activation	Drastic activation	mAP
$K=16$	*88.00 ± 10.30%*	$60.20\pm 16.34\%$	$80.20\pm 12.85\%$	$90.80\pm 6.65\%$	$79.80\pm 4.86\%$
$K=32$	$86.80\pm 12.68\%$	$58.60\pm 14.70\%$	$82.20\pm 11.65\%$	$92.20\pm 7.89\%$	$79.95\pm 5.65\%$
$K=64$	$82.60\pm 12.08\%$	*65.80 ± 13.10%*	*84.80 ± 11.99%*	*93.40 ± 8.71%*	*81.65 ± 5.90%*
$K=128$	$86.00\pm 11.60\%$	$65.40\pm 11.46\%$	$77.80\pm 12.00\%$	*93.40 ± 6.58%*	$80.65\pm 5.45\%$
$K=256$	$86.20\pm 9.66\%$	$62.80\pm 16.66\%$	$83.60\pm 11.02\%$	$89.79\pm 9.14\%$	$80.60\pm 5.38\%$

Italic values indicate the best performance in the corresponding columns

### Performance evaluation of temporal aggregation

The effectiveness of the temporal aggregation can be validated by the following experiment. In addition, the vocabulary size is an important parameter. Intuitively, if the codebook size is too small, the histogram feature may lose the discriminative power, while if the codebook size is too large, the histograms from the same class may not possess enough similarity. Fisher vector encoding, as the most suitable encoding strategy for our application, makes use of GMM to generate the compact dictionary. In this section, FV with different vocabulary sizes is applied on Dataset II, and the classification results are shown in Table 1. Combined feature vector (CF) in “Combination of features” section is employed as the frame-level dynamic feature. Fisher vector brings a substantial increase of classification accuracy when compared with the result of CF in “Validation of dynamic features between frames” section. We try five vocabulary size (denoted as *K*): 16, 32, 64, 128 and 256, and the performance of FV encoding increases initially and then decreases as the vocabulary size grows. When the parameter *K* equals 64, it reaches the peak of the performance. However, $K=128,\,256$ could make the encoding vector too sparse, which is somewhat detrimental to performance.

**Table 2:** Performance comparisons (precision, recall and F-score, in percentage) with several mainstreaming methods on Dataset I

Methods	Precision (%)	Recall (%)	F-score (%)
Shape parameters	$60.91\pm 12.80$	$60.85\pm 10.67$	$59.61\pm 13.52$
Zernike moment	$81.64\pm 6.57$	$82.75\pm 6.89$	$81.63\pm 6.63$
Tree graph	$69.92\pm 9.63$	$67.25\pm 9.43$	$65.59\pm 9.49$
DTW-radial distance	$82.73\pm 11.96$	$81.59\pm 12.28$	$81.40\pm 11.49$
RDOF feature	$82.90\pm 7.25$	$82.50\pm 9.61$	$82.27\pm 5.62$
SAPHIRE	$73.56\pm 8.30$	$74.17\pm 9.97$	$73.36\pm 8.54$
LDP	$90.03\pm 6.89$	$88.13\pm 7.06$	$88.97\pm 7.18$
TBoW	$89.84\pm 5.58$	88.30 ± *6.35*	$88.84\pm 4.00$
VFA (ours)	*93.70 ± 5.10*	*89.70* ± 6.88	*91.41 ± 3.98*

Italic values indicate the best performance in the corresponding columns

**Table 3:** Performance comparisons (precision, recall and F-score, in percentage) with several mainstreaming methods on Dataset II

Methods	Precision (%)	Recall (%)	F-score (%)
Shape parameters	$38.34\pm 14.16$	$45.45\pm 11.35$	$29.91\pm 13.52$
Zernike moment	$55.95\pm 10.16$	$60.65\pm 12.75$	$54.46\pm 11.49$
Tree graph	$57.15\pm 12.36$	$54.40\pm 11.80$	$53.46\pm 10.30$
DTW-radial distance	$61.66\pm 14.25$	$63.95\pm 13.14$	$55.81\pm 13.88$
RDOF feature	$66.37\pm 12.95$	$65.85\pm 17.24$	$64.63\pm 14.31$
SAPHIRE	$58.24\pm 7.86$	$57.65\pm 6.86$	$56.76\pm 7.20$
LDP	$81.72\pm 7.60$	$80.45\pm 8.46$	$79.09\pm 8.05$
TBoW	$80.95\pm 11.79$	$79.95\pm 17.52$	$79.29\pm 12.58$
VFA (ours)	*82.34 ± 4.67*	*81.65 ± 5.90*	*81.27 ± 5.06*

Italic values indicate the best performance in the corresponding columns

### Effectiveness of the proposed framework

Finally, we evaluate the performance of our proposed framework in comparison with several existing algorithms. These algorithms are divided into two groups. On the one hand, The first one corresponds to the first five rows in Tables 2 and 3. Moreover, it contains shape parameters, ZM, TG, Dynamic-Time-Warping-based Radial distance (DTW-Radial distance) as well as radial distance and optical flow combined features (RDOF feature).^4^ [5, 7, 13, 18, 20]. These five algorithms mainly focus on modeling the cell dynamic between frames without emphasizing temporal aggregation. Specifically, a subsequence is sampled from a particular video-clip with fixed frame interval (specified as 20) except that DTW-Radial distance obtains the subsequence using dynamic time warping.^5^ Then cell dynamic features are extracted on the subsequence and concatenated into the video-range feature of cell dynamics. On the other hand, the last four rows in Tables 2 and 3 belong to the other group. In this group, not only the short-term cell dynamics but temporal aggregation are in-depth considered. Stochastic annotation of phenotypic individual-cell responses (SAPHIRE) framework only employs shape parameters as the descriptors of cell shape dynamics, and models video-range cell dynamics with HMM [24]. Local deformation pattern (LDP) framework employs radial distance to characterize cell deformation and accumulates the continuous deformation along the radial direction [21]. Temporal bag-of-word (TBoW) framework was reported in our previous work [25], and our proposed framework is denoted as VFA.

The experiments are conducted on the Dataset I and Dataset II, and the experimental results (classification precision, recall, and F-score measures) are summarized in Tables 2 and 3, respectively. The cell dynamics in Dataset I is categorized into two class, normal and abnormal, while in Dataset II the cell dynamics of abnormal are further annotated as three sub-categorization (slight, moderate and drastic activation). As shown in Tables 2 and 3, RDOF feature achieves a better performance than other methods in group one. It indicates that the dynamic features from cell contour and cytoplasmic streaming are complementary to each other. Compared with DTW-Radial distance, the RDOF feature improves $0.87\%$ F-score in Dataset I, but $8.82\%$ F-score in Dataset II. This illustrates that integrating cytoplasm streaming dynamics brings more improvement for the complex situation, i.e., refined categorization of abnormal cell dynamics.

Because of modeling the video-range temporal dynamics, the frameworks in the second group bring a substantial absolute increase over the corresponding features they used. For example, SAPHIRE benefits $13.75\%$ and $26.85\%$ F-score increases over shape parameters in Tables 2 and 3, separately. Similarly, LDP also obtains a better performance in contrast with DTW-Radial distance. The fact that these two methods obtain better performance on two datasets proves the significance of the temporal aggregation of the cell dynamics. TBoW and VFA are based on the same primary feature (CF in “Combination of features” section), but VFA achieves a better performance ($93.70\%$ precision, $89.70\%$ recall rate and $91.41\%$ F-score in Table 2, $82.34\%$ precision, $81.65\%$ recall rate and $81.27\%$ F-score in Table 3). It manifests that it is wise to introduce the FV encoding for the temporal aggregation of cell dynamics. At last, VFA reaches the peak of performance in both Tables 2 and 3. These results show that our proposed framework outperforms other existing algorithms.

## Discussion

The proposed framework is convenient to extend to other applications about cell temporal dynamics or cell deformation estimation. The whole framework is theoretically compatible with the classification tasks based on cell temporal dynamics. For example, the cellular-response-based drug classification tasks focus on exploring how the cellular response is variation with different drug stimulus, which is able to be captured as cell temporal dynamics in videos. The proposed framework can be considered as a scheme for these tasks. Moreover, part of the framework may also benefit the living cell study. There are some other applications in need of cell temporal dynamics. Modeling the cell cycle, for instance, incorporates the temporal information into the annotation strategy of cellular states in time-lapse movies. The existing methods, in general, exploit the static cell morphology as frame-level features and HMM as feature aggregation strategy. Our frame-level cell dynamic features might serve as the complement of cell morphology feature for cell cycle modeling.

In addition, there are some limits and assumptions in our proposed framework. The shape-context and SIFT flow both assume the time interval between frames should short enough relative to the cell temporal dynamics. And we use SIFT flow to approximately describe 3D cytoplasmic streaming. Although this method is effective for modeling intracellular movement to some extent, we plan to investigate how to model cytoplasmic streaming in 3D space in the future work.

## Conclusion

We have presented a novel framework to evaluate the cell dynamics in video-clips, which first extracts frame-level cell dynamic features based on both contour deformation and cytoplasmic streaming, and then leverages compact encoding to aggregate these short-term features into a video-range cell dynamics. A series of experiments are conducted to evaluate the proposed framework. The first experiment not only verifies the effectiveness of the proposed cell dynamic features, but proves that the MFAFF can more precisely model the cytoplasmic streaming. The second experiment about temporal aggregation figures out the most suitable encoding strategy and its corresponding best parameters. Finally, the proposed framework has been compared with the existing mainstreaming approaches on two datasets, and experimental results show its outperformance in the assessment and classification of cell dynamics.

## Additional file

**Additional file 1: Figure S1.** (a) and (b) correspond to a normal cell and drastic activation cell inDataset II. On the right side, there are two contour sequences. On the left side,blue lines show the radial distance sequences of contour points at 80°, red linesrepresent the smooth sequences; x-axis is time-lapse and y-axis is redialdistance.

## Abbreviations

SIFT: Scale-Invariant Feature Transform
MFAFF: Movement Field and Appearance Change Field Feature
HMM: hidden Markov models
TBoW: temporal bag of words
FV: Fisher vector
VLAD: vector of locally aggregated descriptors
HVLAD: higher-order VLAD
SST: self-skin transplantation
AST: allergenic-skin transplantation
TPS: thin plate spline model
HOSF: histograms of oriented SIFT flow
GMM: Gaussian mixture model
PCA: principal component analysis
SSR: signed square root
SVM: support vector machine
TPP: temporal pyramid pooling
ZM: Zernike moment
TG: tree graph
RDF: radial-distance feature
SCF: shape-context feature
OFF: optical flow feature
SFF: SIFT flow feature
CF: combined feature vector
DTW: dynamic time warping
RDOF: radial distance and optical flow
SAPHIRE: stochastic annotation of phenotypic individual-cell responses
LDP: local deformation pattern
VFA: video feature aggregation

## Footnotes

1 Tree graph is suitable to compute the number and length of cell protrusions.

2 The code for shape context can be found in https://www2.eecs.berkeley.edu/Research/Projects/CS/vision/shape/sc_digits.html.

3 The code of SIFT flow is available in https://people.csail.mit.edu/celiu/SIFTflow/.

4 RDOF feature employs a fusion strategy of multiple features, including radial distance feature and optical flow feature in “Validation of dynamic features between frames” section [18] And the fusion strategy brings an improvement for profiling cell dynamics.

5 Dynamic time warping can obtain a more suitable subsequence for analyzing cell dynamics, which makes DTW-Radial distance achieve a better performance.
