# Supplementary material for: Analyzing temporal dynamics of cell deformation and intracellular movement with video feature aggregation
Source: Biomed Eng Online. 2019 Mar 1;18:20. doi: 10.1186/s12938-019-0638-1 (PMC6397461; doi:10.1186/s12938-019-0638-1)
Supplement: Supplementary file 1 — Additional file 1: Figure S1. (a) and (b) correspond to a normal cell and drastic activation cell inDataset II. On the right side, there are two contour sequences. On the left side,blue lines show the radial distance sequences of contour points at 80°, red linesrepresent the smooth sequences; x-axis is time-lapse and y-axis is redialdistance. [file 12938_2019_638_MOESM1_ESM.pdf]

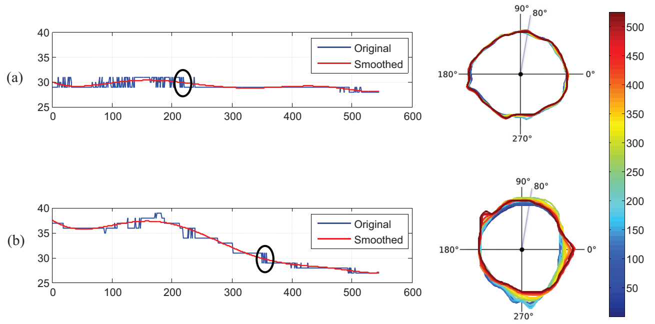

Figure S1: (a) (b) correspond to a normal cell and drastic activation cell in Dataset II. On the right side, there are two contour sequences. On the left side, blue lines show the radial distance sequences of contour points at 80° , red lines represent the smooth sequences; $x - axis$ is time-lapse and $y - axis$ is redial distance.
